# Dr. Horace Tracy Hanks

**Published:** 1900-12

**Authors:** 


Dr. Horace Tracy Hanks, of New York, died at his home, No. 766 Madison Avenue, of nephritis, November 18, 1900, after an illness of one week, aged 63 years. He graduated at Albany Medical College in 1861, served in the Union Army as assistant surgeon with the Thirtieth New York Volunteers, was gynecologist at the Demilt Dispensary from 1872 to 1882; assistant surgeon at the Woman's Hospital from 1875 to 1879, and surgeon since the latter year. Since 1885 he had been professor of diseases of women in the New York Post-Graduate Medical School and Hospital.

